# Rabies: Knowledge, Attitude and Practices in and Around South Gondar, North West Ethiopia

**DOI:** 10.3390/diseases8010005

**Published:** 2020-02-24

**Authors:** Amare Bihon, Desalegn Meresa, Abraham Tesfaw

**Affiliations:** 1 School of Veterinary Medicine, Woldia University, Woldia 7220, Ethiopia; 2 College of Health Science, Mekele University, Mekele 7000, Ethiopia; desalegnmeresa21@yahoo.com; 3 College of Veterinary Medicine, Samara University, Samara 7240, Ethiopia; Abrahamtesfaw2009@gmail.com

**Keywords:** south Gondar, attitude, factors, knowledge, practice, rabies

## Abstract

A cross-sectional study was conducted from February 2017 to April 2017 to assess knowledge, attitude and practices of the community towards rabies in south Gondar zone, Ethiopia. A structured closed ended questionnaire was used to collect the data through face to face interviews among 384 respondents. The data were then analyzed using SPSS statistical software version 20. Almost all (91.5%) surveyed individuals were aware of rabies. Bite was known as mode of rabies transmission by majority of the respondents (71.1%) with considerable means of transmission through wound contact with saliva of diseased animals. Sudden change of behavior was described as a major clinical sign of rabies in animals by the majority of the respondents. Nearly half of the respondents (48.2%) believed that consumption of rabid animal’s meat can be a medicine for human rabies and majority of the respondents (66.7%) indicated crossing a river before 40 days after dog bite increases severity of the disease. More than eighty percent of the respondents prefer traditional medicines for treating rabies in humans. In total, 51% of the respondents had poor Knowledge, Attitudes and Practices (KAP) level about the disease rabies. Educational status (*χ*^2^ = 21.152), Monthly income (*χ*^2^ = 23.059), Sex (*χ*^2^ = 11.249), source of information (*χ*^2^ = 8.594) and Residence (*χ*^2^ = 4.109) were significantly associated with KAP scores (*p* < 0.05). Education and awareness creation should be given to increase communities KAP about the disease with special focus to traditional healers.

## 1. Introduction

The word rabies originates from the Latin word rabere. Rabere means to rage or rave, and may have roots in a Sanskrit word rabhas, which means to do violence [1]. Rabies was present in Egypt before 2300 B.C and also in ancient Greece [2]. The association between animal bites and human fatal disease has been recognized in many parts of the world for more than 2000 years. Rabies ranked 12th on the WHO list of major killer diseases [3] and it is under OIE (Office International des Epizootics) category of multiple species diseases, infections and infestations. Currently, it remains an ongoing threat to human populations and animals in many parts of the world [4,5,6]. The etiologic agent of this disease is the rabies virus belonging to the genus *Lyssa virus* and family Rhabdoviridae [7]. Rabies is one of the most serious zoonotic diseases. Once the clinical signs developed, it is almost 100% fatal disease [8,9]. 

Next to Asia, Africa is the most affected continent by rabies with an estimated 24,000 (44%) of the 55,000 annual rabies deaths worldwide [10], which increases to 59,000 annual rabies deaths after 10 years [11,12,13]. Domestic dogs are considered to be the main sources (>90%) of human rabies in Africa [14] and more than 88% of the exposure cases in Ethiopia were due to dog bites [15]. Canine rabies is endemic in Ethiopia [13], with an estimated 2771 human deaths annually [12,16]. A national surveillance conducted in Ethiopia from 2007 to 2012 showed that 15,178 exposure cases (3.4/100,000) and 272 fatal cases with 1.5 incidence rates in every 100,000 people living in the Amhara region [15]. More specifically, in the study area, an incidence rate of 7.5 in every 100,000 people was reported in 2016 by [17]. Annual rabies-associated livestock losses in the world are estimated at >$50 million (USD), making rabies important to both human and animal health [12,18].

Prevention and control can be achieved through strict quarantine measures, elimination of stray dogs, extension program, control of rabies in wild life, registration of dogs and prophylactic vaccination [19]. Human rabies deaths are almost entirely preventable through prompt delivery of post-exposure prophylaxis (PEP) to victims bitten by rabid animals [20,21]. Rabies victims especially from rural areas seek PEP treatment after exhausting the traditional medicinal intervention and usually after loss of a family member [22,23,24,25]. Nervous tissue vaccine (sheep brain derived Fermi type vaccine), cell culture based vaccines and rabies immunoglobulin (RIG) are available in Ethiopia but only fermi type vaccine is available in the study area with limited access [26,27,28,29]. 

In Ethiopia, few studies have been conducted to assess community awareness towards rabies [30,31,32], which are concentrated in Addis Ababa due to the presence of large population of stray dogs [32,33]. Poor public awareness towards rabies is considered as one of the bottle necks for the prevention and control of the disease in Ethiopia especially in rural areas. Therefore, this study was designed to assess the level of Knowledge, Attitudes and Practices (KAP) of the community towards rabies in south Gondar zone, Ethiopia.

## 2. Material and Methods

### 2.1. Study Area

South Gondar Zone is found in Amhara Regional state of Ethiopia. It is named for the city of Gondar, which was the capital of Ethiopia until the mid-19th century, and has often been used as a name for the local province. South Gondar is bordered on the south by East Gojjam, on the southwest by West Gojjam and Bahir Dar, on the west by Lake Tana, on the north by North Gondar, on the northeast by Wag Hemra, on the east by North Wollo, and on the southeast by South Wollo. The highest point in South Gondar is Mount Guna (4231 m). It comprises 15 districts (Debre Tabor, Dera, Ebenat, Farta, Fogera, Guna Begemier, Kemekem, Lay Gayint, Mena Meketewa, Mirab Este, Misraq Este, Sede Muja, Simada, Tach Gayint and Woreta). The climate of the area is Woina dega with 2077 meters above sea level. The area has 1300 mm^3^ mean annual rain fall and 26 °C mean annual temperature.

### 2.2. Study Population, Design and Sampling Technique

The target groups for the interview were household heads who lived in south Gondar zone. A cross-sectional study design and multi-stage sampling procedure were employed to select households for this study. Three districts (Dera, Ebnat and Debretabor) were randomly selected using lottery method from list of districts in south Gondar zone, followed by selection of 128 household heads from each district using systematic random sampling method by considering every 10th households. Whenever the selected household was found locked, the next household (on the right side) was substituted automatically for interview. To reduce the risk of households locked, we prefer Sunday, which is a religiously respected day and people stay at home.

### 2.3. Sample Size Determination

The sample size was calculated using the formula for estimating single population proportion. The assumptions used were: 95% confidence interval, a marginal error of 0.5 and the proportion of awareness level of 50% [34]. Then the required sample size 384 was computed. 

### 2.4. Data Collection Method

Data was collected using pre-tested interviewer administered structured questionnaire. The questionnaire was developed based on the information gathered from literatures and on what the community is practicing. The questionnaire was first prepared in English and later translated to Amharic (National working language). The interviewer administered the questionnaires through face to face interviews. 

### 2.5. Data Quality Assurance

Before beginning the full study, the pre-test was performed in some participants to see the applicability of the questionnaire. Each questionnaire was checked for incompleteness, missed values and unlikely responses, and then manually cleaned upon such indications. The data was cross checked for consistency and accuracy.

### 2.6. Data Analysis

After collecting the data from the participant using recording format developed for this purpose, the data was cleaned and checked for completeness. Then, the data from the questionnaires were entered into Epi-info 2008 version 3.5.1 software for data cleaning and were transferred to SPSS 20 for analysis. Then after, Descriptive and Chi-squire analysis were performed. KAP scoring was done by modifying the method described by [35] by giving “1” for correct answers and “zero” for wrong and unknown answers. Respondents who scored greater than or equal to the mean value (Mean = 13.35) was grouped as having good KAP and the score less than the mean value was considered as Poor KAP level. Statistical significance was considered at *p*-value less than 0.05.

## 3. Results

### 3.1. Socio-Demographic Characteristics

From a total of 384 respondents, 71.4% were males and greater numbers of the respondents were in the range of 30–45 years of age. Nearly half of the respondents (47.7%) were illiterate and majority of the study participants were married 294 (76.6%). More than seventy percent of the respondents were farmers and 44.5% of the respondents earn monthly income of 31–62 USD (Table 1).

### 3.2. Knowledge, Attitude and Practice Towards Rabies Disease and Its Control and Prevention

Majority of the respondents (77.9%) were aware of the disease rabies. More than half of the respondents knew rabies by different local names like ‘*Yewusha Kelebat*’, ‘*Yebed wusha beshata*’ meaning ‘*mad dog*’. More than eighty percent of the respondents knew fatal nature of the disease once the clinical signs appeared. Almost all (91.9%) of the respondents relied on informal sources of information. The majority of the respondents were aware of rabies indiscriminately affects both sexes and all age groups (Table 2).

### 3.3. Knowledge of Study Participants Related to Transmission and Clinical Sign of Rabies

Majority of the respondents were aware about zoonotic nature of the disease. Bite was known as mode of rabies transmission by majority of the respondents (71.1%) with considerable means of transmission through wound contact with saliva of diseased animals. Sudden change of behavior was described as a major clinical sign of rabid animals by the majority of the respondents (Table 3).

### 3.4. Knowledge *of the Study Participants about Control and Prevention of Rabies*

Majority of the respondents (60.4%) were not aware about post exposure prophylaxis for rabies while 65.9% of the respondents were aware of dog vaccination as a means of rabies prevention. The greater numbers of the respondents were willing to use rabies vaccine for their pets (Table 4). 

### 3.5. Attitude of the Study Participants Towards Rabies

Nearly half of the respondents (48.2%) believed consumption of rabid animal’s meat could be a medicine for rabies. Majority of the respondent’s preferred traditional medicine by the healers than health professional’s advice for treating rabies in humans. Majority of the respondents (66.7%) indicated crossing a river before 40 days after dog bite increases severity of the disease (Table 5).

### 3.6. Practice of the Study Participants Towards Rabies 

Considerable numbers of the respondents own dogs and most of the dogs were free ranging. Fewer numbers of the respondents use rabies vaccine for their dogs. Majority of the respondents practiced killing of rabid animals and seeks traditional healers when humans are bitten by rabid animals. Fewer numbers of respondents practiced washing of wound with soap and water after dog bite (Table 6).

### 3.7. Factors Associated with Community KAP Towards Rabies

More than half of the respondents had poor KAP level. Being sex, educational status, sources of information and monthly income significantly associates with KAP level of the respondents (*p* < 0.05). (Table 7).

## 4. Discussion

### 4.1. General KAP of the Study Participants Towards Rabies

In this study, the majority of the respondents (58.6%) were heard about rabies. This finding is much lower than the findings of different studies in different countries from different community members [36,37,38,39,40]. However, it was higher than the reports of [41] in Kenya, [42] in Tanzania and [43] in Guatemala who reported knowledge rates of 49%, 27% and 0% respectively. These differences could be associated with awareness level of the community, educational status and information access. This indicates the gap of awareness in the study area. 

In this study, 54.7% of the respondents mentioned the host range of the disease as humans and other domestic animals can be infected by rabies. This result was consistent with [37] from Gondar Zuria district and [44] from Dessie city in Ethiopia while the finding in this study was much lower. This suggests consideration of all domestic animals and humans in rabies prevention and control programs. More than ninety percent of informants in this study obtained information from informal sources. Similarly, respondents from Addis Ababa city indicated informal sources as a major sources information [30]. This might be due to frequent contact of the community each other and culture of discussing issues happening in their locality.

Change in behavior (Aggression) was mentioned as a major clinical sign by majority of the respondents which is in line with the fact that furious form of rabies is the common type of rabies in animals [45,46]. Similar but much lower reports about clinical signs were reported from Debark woreda, Ethiopia by [47]. This form of rabies is more easily identifiable clinical form by most people and attracts individuals’ attention as the animals tend to attack humans and other animals. Rabies is known as “mad dog” in the community, which is associated with the clinical sign, aggression. The paralytic form may be overlooked by the community since it may not be noticed by those who associate rabies only with madness. Under such condition, people may not seek post exposure prophylaxis when exposed to animals with the paralytic form of the disease and thus may risk death by rabies. In the present study, more than seventy percent of the respondent’s replied sudden change of behavior and salivation as the clinical signs of rabid dog. This suggests more risk of people by rabid animals with paralytic forms of rabies as the community have religious responsibility to help diseased animals. 

In this study, bite was mentioned as mode of transmission for rabies to humans by the majority of the respondents which is consistent with the findings of [36] and Considerable percent of participants mentioned contact of open wound with saliva as mode of transmission. Inhalation and scratch with teeth were also considered as means of transmission by a few of the respondents. Inoculation of infected saliva through the bite of a rabid animal appears to be the predominant mode of rabies transmission [8]. It is under special conditions have infections occurred through aerosols, (e.g. in caves inhabited by bats and by exposure in the laboratory) [48,49]. Even though the extent of transmission varies, all possible modes of transmission including bite and contact with saliva of diseased animal should be avoided. 

More than half of the respondents indicated regular vaccination of dogs as effective measure for the control of rabies but depopulation of stray dogs were reported as effective control measure by [50] from Bahir dar, Ethiopia and [51] from Sir lanka. Both vaccination and depopulation will play pivotal role in prevention and control programs, while in this community, vaccination might be preferable. Only 39% of respondents are aware of post exposure prophylaxis. Similar study from Delo district of Jimma zone in Ethiopia reported 65.5% awareness level of the community about post exposure prophylaxis. Most of the respondents (81.5%) in this study believed on traditional healers. Similarly, reference [13] reported 84% reliance of respondents on traditional treatment. Also, studies conducted in Addis Ababa, Ethiopia, reported strong beliefs of the participants on traditional healers by 58.3 % of the participants [30]. Reference [44] in and around Dessie city, reference [52] in Araba Minch and [53] from Abia state of Nigeria, all reported the reliance of the community on traditional practices. The preference for traditional practices might arise from many factors including easy access to traditional medicine and lack of awareness. Reliance on traditional medicines with unproven efficacy is very risky in that nothing can be done to save one’s life after the first symptoms of the disease occurred. This awareness gap increases the risk of the disease in the community. So, more efforts should be taken for awareness creation about the importance of post exposure prophylaxis and the risk of traditional treatments. 

In the current study, only 76 (19.8%) of the study participants used rabies vaccine for their dogs while more than 70% of the respondents volunteer to use the vaccine for their pets. Similarly, reference [54] reported little usage of rabies vaccine in Kisumu and Siaya provenience of Kenya. This indicates the gap between supply and demand of rabies vaccine. 

Only fewer (8.9%) numbers of the participants had practiced washing of the wounds with soap and water as first aid to prevent rabies. Other published surveys have indicated that similar proportion of people felt that washing the wound with soap and water was the best option [37,55,56]. Washing of rabies-infected wounds with soap and water can increase survival by 50% [8]. This treatment is cheap, readily available and feasible for all to apply but it needs the greatest effort of stakeholders to adapt it in the community.

Surprisingly, the community believes crossing the river within 40 days of dog bite increases the severity of the disease in humans. It might be associated with the incubation period of the disease and hydrophobia of people infected with rabies virus. Moreover, it indicates the huge perception gap in functional treatments and traditional beliefs.

### 4.2. Factors Associated with Community KAP Towards Rabies

Informal sources were the leading sources of information in the study area (*p* < 0.05). Similar findings were reported by [30] from Addis Ababa. This can be concluded that informal sources play a significant role in disseminating information about rabies and its zoonotic importance to the local community. KAP of the community was significantly associated with monthly income of the households (*p* < 0.05). This finding agrees with the reports of [50] from Bahirdar town. This might be partly explained by the strong social interactions of people having low income in their day to day life which may help them to exchange information.

Sex was significantly associated with KAP level (*p* < 0.05) with higher KAP level in males. Similar findings were reported by [47] in Debark Woreda, North Gondar, Ethiopia. This difference might be due to increased activity of males outside and the culture that limits the movement of females to stay outside their home. Education status was significantly associated (*p* < 0.05) with KAP level of the community, while illiterates led good KAP level. On the contrary, references [50,57] reported higher KAP level in literates having first degree and above. This might be due to way of living, social interaction and way of information exchange.

Residence showed significant association with level of KAP (*p* < 0.05) with higher KAP level in rural community. Similar findings were reported from Delo district of Jimma zone by [57]. This could be due to better communication of the rural community about what is happening in their residential area including animal disease situations which might contribute to their high level of awareness. 

## 5. Conclusions and Recommendations

Rabies is a well-known disease in the study area and most of the study participants prefer traditional treatments by healers for the treatment of human rabies. The majority of the respondents believed consumption of rabid animal’s meat can be a medicine for human rabies and crossing a river before 40 days after dog bite increases the severity of the disease. In total, 51% of the respondents had poor KAP level about the disease rabies. The differences in KAP score among participants were dependent on sex, educational status, source of information, monthly income, and residence. Therefore, periodic education should be given to raise community KAP on rabies and more emphasis should be given for traditional practices and healers. 

## Figures and Tables

**Table 1:** Socio-demographic characteristics of the study participants (N = 384), 2017.

Socio-Demographic Variable	Frequency (%)
Sex	
Male	274 (71.4)
Female	110 (28.6)
Age ≤ 29	58 (15.1)
30–45	180 (46.9)
≥46	146 (38)
Family size	
≤3	127 (33.1)
4–6	193 (50.3)
≥7	64 (16.7)
Income per month	
≤30 USD	151 (39.3)
31–62 USD	171 (44.5)
≥63 USD	62 (16.1)
Education	
Illiterate	183 (47.7)
Basic	115 (29.9)
Primary and secondary	58 (15.1)
Above secondary	28 (7.3)
Residence	
Urban	72 (18.8)
Rural	312 (81.3)
Marital Status	
Married	294 (76.6)
Unmarried	31 (8.1)
Others	59 (15.4)
Living situation	
With family	346 (90.1)
Alone	38 (9.9)
Occupation	
Farmer	282 (73.4)
Merchant	23 (6)
Health professional	1 (0.3)
Others	78

NB: others in occupation (teachers, employees, government officials) and others in marital status (widowed and divorced).

**Table 2:** General knowledge of the study participants towards rabies (N = 384), 2017.

Knowledge of Rabies Disease	Frequency (%)
Have you heard about rabies?	
Yes	299 (77.9)
No	85 (22.1)
What is rabies?	
Disease	225 (58.6)
Change in behavior	54 (14.1)
Do not know	105 (27.3)
Species affected by rabies	
Dog only	42 (10.9)
Dog and human	132 (34.4)
Human and other domestic animals	210 (54.7)
Easily treated after onset of clinical sign	
Yes	68 (17.7)
No	316 (82.3)
Which age group is mostly affected	
Young	59 (15.4)
Adult	7 (1.8)
Male	2 (0.6)
Female	9 (2.3)
All	307 (79.9)
Source of information about rabies	
Formal	31 (8.1)
Informal	353 (91.9)
Fate of a person or animal bitten by rabid dog	
Die	325 (84.6)
Carrier	2 (0.5)
Nothing happen/cure	28 (7.3)
I don’t know	29 (7.6)
Part of the body affected by rabies	
Brain	167 (43.5)
Stomach	85 (22.1)
Bitten area	43 (11.2)
Brain and stomach	8 (2.1)
I don’t know	81 (21.1)

**Table 3:** Knowledge of the study participants about transmission and clinical sign of rabies (N = 384), 2017.

Knowledge Related Variables	Frequency (%)
Transmitted from rabid animal to healthy animal	
Yes	228 (59.4)
No	156 (40.6)
Transmitted from rabid animal to human	
Yes	215 (56)
No	169 (44)
Mode of transmission from rabid animal to healthy animals/human	
Biting	273 (71.1)
Inhalation	2 (0.5)
Scratch	3 (0.8)
Saliva contact with open wound	218 (56.8)
Clinical signs of rabid dog	
Salivation	272 (70.8)
Sudden change of behavior	291 (75.8)
Stop eating and drinking	129 (33.6)
Hydrophobia	21 (5.5)
Paralysis	5 (1.3)
Hallucination	17 (4.4)

**Table 4:** Knowledge of the study participants for prevention and control towards rabies (N = 384).

Knowledge Related Variables	Frequency (%)
Do you know about post exposure prophylaxis?	
Yes	152 (39.6)
No	232 (60.4)
Can we prevent rabies by vaccination?	
Yes	253 (65.9)
No	131 (34.1)
Do you know the vaccination interval for pets?	
Yes	108 (28.1)
No	276 (71.9)
Do you think rabies vaccine is important?	
Yes	262 (68.2)
No	122 (31.8)
Are you willing to vaccinate your pet?	
Yes	268 (69.8)
No	116 (30.2)

**Table 5:** Attitude of the study participants towards rabies (N = 384), 2017.

Attitude Related Variables	Frequency (%)
consumption rabid animal meat could be a medicine for rabies	
Agree	185 (48.2)
Do not agree	172 (44.8)
I don’t know	27 (7)
Burning the rabid animal and inhalation of the smoke could be a medicine for rabies	
Agree	0 (0.0)
Do not agree	197 (51.3)
I don’t know	187 (48.7)
Traditional healers could be a solution for rabies	
Agree	313 (81.5)
Do not agree	55 (14.3)
I don’t know	16 (4.2)
Crossing a river before 40 days could prevent disease development	
Agree	42 (10.9)
Do not agree	256 (66.7)
I don’t know	86 (22.4)
Do you seek health professional advice if you are bitten by rabid animal?	
Yes	79 (20.6)
No	305 (79.4)

**Table 6:** Practices of the study participants towards (N = 384), 2017.

Practice Related Variables	Frequency (%)
Dog ownership	
Yes	163 (42.4)
No	221 (57.6)
Dog management	
Indoor	154 (40.1)
Free	230 (59.9)
Action to be taken on animals bitten by rabid dog	
Killing	91 (23.7)
No action	118 (30.7)
Treatment	175 (45.6)
Immediate Action taken on rabid animal	
Tie	134 (34.9)
Kill	247 (64.3)
Nothing	3 (0.8)
Immediate action taken after bite of humans by rabid animal	
Washing with soap and water	34 (8.9)
Use traditional healer	224 (58.7)
Visit health institution	117 (30.5)
Do nothing	22 (5.7)
Have you vaccinated your dog for rabies?	
Yes	76 (19.8)
No	308 (80.2)
Purpose of owning dog	
Guarding	373 (97.1)
Hunting	5 (1.3)
Guarding and hunting	6 (1.6)
Family history of rabies infection	
Yes	144 (37.5)
No	240 (62.5)
Have you ever bitten?	
Yes	132 (34.4)
No	252 (65.6)

**Table 7:** Factors associated with Knowledge, Attitudes and Practices (KAP) of participants towards rabies (N = 384), 2017.

Variables	Poor KAP	Good KAP	*χ* ^2^	*p*-Value
Age				
≤29	28 (7.3%)	30 (7.8%)	0.238	0.888
30–45	92 (24%)	88 (23%)		
≥46	76 (19.8%)	70 (18.2%)		
Sex				
Male	125 (32.6%)	149 (38.8%)	11.249	0.001
Female	71 (18.4%)	39 (10.2%)		
Residence				
Urban	29 (7.6%)	43 (11.2%)	4.109	0.043
Rural	167 (43.4%)	145 (37.8%)		
Marital status				
Married	142 (37%)	152 (39.6%)	4.465	0.215
Unmarried	18 (4.7%)	13 (3.4%)		
Others	36 (9.3%)	23 (6%)		
Education				
Illiterate	106 (27.6%)	77 (20%)	21.152	0
Basic	62 (16.1%)	53 (13.8%)		
Primary and secondary	24 (6.3%)	34 (8.9%)		
Above secondary	4 (1%)	24 (6.3%)		
Occupation				
Farmer	146 (38%)	136 (35.4%)	1.785	0.618
Merchant	13 (3.4%)	10 (2.6%)		
Animal and human Health professional	0 (0%)	1 (0.3%)		
Others	37(9.6%)	41 (10.6%)		
Living situation				
With family	174 (45.3%)	172 (44.8%)	0.793	0.373
Alone	22 (5.7%)	16 (4.2%)		
Monthly income				
≤30 USD	100 (26%)	53 (13.8%)	23.059	0
31–62 USD	76 (19.8%)	95 (24.7%)		
≥63 USD	20 (5.2%)	40 (10.4%)		
Family size				
≤3	74 (19.3%)	53 (13.8%)	4.437	0.109
6–4 month	94 (24.5%)	99 (25.8%)		
≥7	28 (7.3%)	36 (9.4%)		
Source of information				
Formal	8 (2%)	23 (6%)	8.594	0.003
Informal	188 (49%)	165 (43%)		
Family history of rabies infection				
Yes	60 (15.6%)	84 (21.8%)	10.161	0.017
No	136 (35.4)	104 (27.1%)		
Have you ever bitten				
Yes	62 (16.1%)	70 (18.2%)	1.335	0.248
No	134 (34.9%)	118 (30.7%)		
Total	196 (51%)	188 (49%)		

NB: Occupation: Others (teachers, employees, government officials)
